# IL-27 inhibits the TGF-β1-induced epithelial-mesenchymal transition in alveolar epithelial cells

**DOI:** 10.1186/s12860-016-0084-x

**Published:** 2016-03-01

**Authors:** Zhaoxing Dong, Wenlin Tai, Wen Lei, Yin Wang, ZhenKun Li, Tao Zhang

**Affiliations:** Department of Respiratory, The 2nd Affiliated Hospital of Kunming Medical University, Dianmian Road 374, Kunming, Yunnan 650101 China; Department of Clinical Laboratory, Yunnan Molecular Diagnostic Center, The 2nd Affiliated Hospital of Kunming Medical University, Dianmian Road, Kunming, Yunnan China

**Keywords:** Interleukin 27, Epithelial–mesenchymal transition, Alveolar epithelial cells, Signalling pathway

## Abstract

**Background:**

IL-27 is a multifunctional cytokine that has both pro-inflammatory and anti-inflammatory functions. Although IL-27 has been shown to potently inhibit lung fibrosis, the detailed mechanism of IL-27 in this process is poorly understood. Epithelial–mesenchymal transition (EMT) is one of the key mechanisms involved in pulmonary fibrosis. We assessed the effects of IL-27 on TGF-β1-induced EMT in alveolar epithelial cells.

**Methods:**

A549 cells (a human AEC cell line) were incubated with TGF-β1, IL-27, or both TGF-β1 and IL-27, and changes in E-cadherin, β-catenin, vimentin and a-SMA levels were measured using real-time PCR, western blotting and fluorescence microscopy. The related proteins in the JAK/STAT and TGF-β/Smad signalling pathways were examined by western blot.

**Results:**

IL-27 increased the expression of epithelial phenotypic markers, including E-cadherin and β-catenin, and inhibited mesenchymal phenotypic markers, including vimentin and a-SMA in A549 cells. Moreover, TGF-β1-induced EMT was attenuated by IL-27. Furthermore, we found that TGF-β1 activated the phosphorylation of JAK1, STAT1, STAT3, STAT5, Smad1, Smad3 and Smad5, and IL-27 partially inhibited these changes in this process. When cells were treated with the STAT3 specific inhibitor wp1006 and the Smad3 specific inhibitor SIS3, the inhibition of EMT by IL-27 was significantly strengthened.

**Conclusion:**

Our results suggest that IL-27 attenuates epithelial–mesenchymal transition in alveolar epithelial cells in the absence or presence of TGF-β1 through the JAK/STAT and TGF-β/Smad signalling pathways.

**Electronic supplementary material:**

The online version of this article (doi:10.1186/s12860-016-0084-x) contains supplementary material, which is available to authorized users.

## Background

Pulmonary fibrosis is characterized by the destruction of lung tissue architecture and the formation of fibrous foci. Some studies suggested that pulmonary fibrotic diseases included a three phase model of wound repair-injury, inflammation and repair [1]. A subsequent hypothesis suggested that epithelial injury and impaired wound repair, without preceding inflammation, were the aetiology of fibrosis [2]. Mounting evidence suggests that one possible mechanism of fibrotic disease pathogenesis involves alveolar epithelial cell (AEC)-derived fibroblasts through epithelial–mesenchymal transition (EMT) [3, 4]. Although research has made advances in unveiling the molecular mechanism of pulmonary fibrosis, current treatments for idiopathic pulmonary fibrosis show poor efficacy and do not prevent or reverse the disease progression [5].

IL-27 is a heterodimeric cytokines that includes EB virus-induced gene 3 (EBI3) and P28 (IL-27p28) and plays an important role in T cell differentiation. IL-27, by inhibiting the expression of the RORγt master transcription factor, prevented the development of proinflammatory Th17 cells and inhibited the production of IL-17A and IL-17 F in naive T cells [6]. The IL-27 receptor is made up of gp130 and WSX-1, and associates with cytoplasmic protein kinases, such as JAKs (Janus Activated Kinases) that mediate cytokine signalling [7]. The JAK/STAT signalling pathway was initially identified as a critical pathway for normal cellular processes but has also been implicated in pulmonary fibrosis [8]. Our previous work demonstrated that IL-27 might inhibit Th17 cell differentiation and the secretion of related inflammation factors in a bleomycin-induced pulmonary fibrosis model [9].

AECs are important target cells that can directly promote lung fibrosis by acquiring a mesenchymal phenotype through EMT. EMT is genetically characterized by a decreased expression of epithelial cell-associated genes (E-cadherin) and increased expression of mesenchymal cell-associated genes, such as α-smooth muscle actin (α-SMA) [10, 11]. Recent studies demonstrated the role of EMT in pulmonary fibrosis [3, 12]. The TGF-β/Smad signalling pathway is required for both EMT and fibrosis in a variety of organs [13].

Currently, the role of IL-27 in idiopathic pulmonary fibrosis is not clearly defined. Shen [14] found that IL-27 might be involved in DM and PM pathogenesis. Moreover, higher levels of IL-27 were measured in patients with interstitial lung disease (ILD). Given these results, we hypothesized that IL-27 may be involved in lung fibrosis. We previously established that IL-27 is involved in pulmonary fibrosis in a bleomycin-induced mouse model, but the specific mechanism was not determined. In this study, we identified potential molecular mechanisms of the effects of IL-27 on pulmonary fibrosis. We found that treatment of A549 cells with IL-27 inhibited EMT-related changes and attenuated the effects of TGF-β1.

## Methods

### Cell culture

A549 cells were purchased from the Kunming Animal Institute and cultured in complete medium containing Dulbecco’s Modified Eagle’s Medium (DMEM) with high levels of glucose and L-glutamine supplemented with 10 % (v/v) foetal bovine serum (FBS) and 1 % (v/v) antibiotic/antimycotic agents (all from Invitrogen Canada, Inc., Burlington, ON, Canada) and maintained in 5 % CO_2_ at 37 °C. All procedures were performed in accordance with the Declaration of Helsinki of the World Medical Association. Additionally, the protocols were approved by the IRB/Ethics Committee of Kunming Medical University. For IL-27 and TGF-β1 cytokines used in this research were recombinant mouse cytokines from eBiosience(San Diego, California, USA).

### MTT assay

Cell viability was measured in a quantitative colorimetric MTT assay (Beyotime, Nantong, China). Briefly, cells were seeded in 96-well plates (6 × 10^3^ cells/well) and maintained in growth media for 24 h with 5 % CO_2_ at 37 °C. When the cells reached 60 % confluence, they were treated with different concentrations of IL-27 or TGF-β1 for 48 h. Next, 10 μl of the MTT solution (5 mg/ml) was added to each well, and the cells were incubated for another 4 h at 37 °C. After formazan crystals formed, the MTT medium was aspirated and replaced with 150 μl of dimethyl sulfoxide (DMSO) (Beyotime, Nantong, China) to solubilize the crystals. Then, the plates were shaken for 5 min. The absorbance of each well was recorded using a microplate spectrophotometer at 570 nm. Relative cellular growth was determined by the ratio of the average absorbance of treated cells versus the average absorbance of control cells. Cell viability was calculated as the ratio of the optical densities.

### Real-time quantitative RT-PCR

RNA was obtained from cultured fibroblasts using TRIzol Reagent (TaKaRa, Japan) according to the manufacturer’s protocol. RNA was then reverse transcribed using a Prime Script RT Reagent Kit (TaKaRa). The total RNA (1 μg) from each tissue sample was reverse-transcribed to cDNA as follows: 8 μl of 5X Prime Script Buffer (for real-time); 2 μl of Prime Script RT Enzyme Mix; 0.1 nmol oligo(dT) primer; 0.2 nM random hexamers; 2 μg of total RNA; and RNase-free deionized water to a final volume of 40 μl. The reverse transcription proceeded for 15 min at 37 °C and 5 s at 85 °C. The specific primers were designed using Primer Premier 5.0. All primers were synthesized by Sangon Biotechnology. An ABI 7300 Real-Time PCR System (ABI, USA) was used for RT-PCR amplification and detection. RT-PCR reactions were prepared in triplicates in 20-μl reaction volumes as follows: 10 μl of 2X SYBR Premix Ex Taq II, 0.4 μM of forward and reverse primers, 2 μl of cDNA template, and 6.4 μl of RNase-free water. Master Mix without cDNA template was used as a negative control. RT-PCR cycling conditions were used as suggested in the SYBR Premix Ex Taq II Kit instructions (TaKaRa, Japan). Melting curves were evaluated to ensure the specificity of the PCR products in the SYBR Green reactions. Relative mRNA levels of the target genes were normalized to β-actin mRNA. The following oligonucleotide primers specific for human genes were used. a-SMA, 5′-CGGGACATCAAGGAGAAACT (sense) and 5′-CCCATCAGGCAACTCGTAA-3′(antisense); E-cadherin, 5′-ATGCTGAGGATGATTGAGGTGGGT-3′(sense) and 5′-CAAATGTGTTCAGCTCAGCCAGCA-3 (antisense);β-catenin, 5′-TGCAGTTCGCCTTCACTATGGACT-3′(sense) and 5′-GATTTGCGGGACAAAGGGCAAGAT-3′ (antisense); Vimentin, 5′-AGAACC TGCAGGAGGCAGAAGAAT-3′(sense) and 5′-TTCCATTTCACGCATCTGGCGTTC -3′(antisense); β-actin, 5′-TGACGTGGACATCCGCAAAG-3′ (sense) and CTGGAA GGTGGACAGCGAGG-3′(antisense).

### Western blot

Total protein concentration was measured using a BCA Protein Assay Kit (Beyotime, ShangHai, China). For western blotting, 30 μg of protein was loaded into each lane of 10 % SDS PAGE gels and was followed by electrophoresis and protein transfer to PVDF membranes (Millipore). After the transfer, the membranes were blocked with 5 % BSA in PBST. Immunoblots were probed with primary antibody at 4 °C overnight followed by secondary antibodies (Proteintech, 1:5000 dilution) for 30 min at room temperature. After extensive washing, the membranes were incubated in ECL reagent (Pierce, Thermo Co., Ltd, USA) for HRP detection and then exposed to autoradiography film (Bio-Rad, Co., Ltd, USA) for band visualization. β-Actin was used as a loading control. The relative amounts of various proteins were analysed, and the results were quantified using Image J software.

### Immunofluorescence staining and fluorescence microscopy

Cells were grown in 6-well glass-bottomed dishes. After the cells were treated, they were fixed in 4 % paraformaldehyde for 30 min and then permeabilized with 0.2 % Triton X-100 in PBS. Non-specific binding sites were blocked with normal goat serum (Sigma-Aldrich, USA) diluted in 0.1 % Triton-X-100 in PBS for 2 h. Then, the cells were incubated overnight at 4 ° C with primary antibodies at a 1:200 dilution in blocking buffer. Primary antibodies were purchased from Proteintech Technology Company. The next day, the cells were incubated with appropriate fluorescein-conjugated secondary antibodies. DAPI was used to stain nuclei before acquiring images. The images were acquired using a fluorescence microscope (Olympus, Tokyo, Japan); the green or red fluorescence indicated positive antibody expression, and the blue fluorescence was nuclear DAPI labelling. The labelled fields of each section were analysed to produce a mean optical density value (MOD), which represents the strength of the staining signals as measured per positive pixel.

### Statistical analysis

Data are presented as the mean response ± S.E.M ($\overline{\chi}\pm s$) and analysed using GraphPad Prism 5.0 software to compare mean values between groups in a one-way ANOVA and Tukey’s test, *P < 0.05, **P < 0.01, ***P < 0.001.

## Results

### IL-27 affects A549 cells in a concentration-dependent manner

**Fig. 1:**
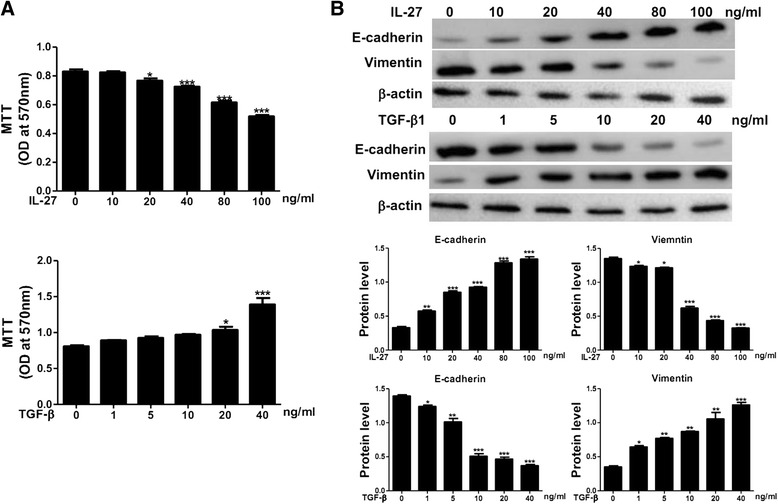
Effects of IL-27 and TGF-β1 on A549 cells. **a** A549 cells were treated with various concentrations of IL-27 for 48 h. Cell proliferation was analysed in an MTT assay. **b** A549 cells were treated with various concentrations of IL-27 and TGF-β1. Protein levels of E-cadherin and vimentin were measured by a western blot. For subsequent experiments 100 ng/ml of IL-27 and 40 ng/ml of TGF-β1 were used. Values are expressed as the mean ± SD from at least three experiments. Statistical significance was assessed by one-way ANOVA and Tukey’s post hoc test. Compared with control *P < 0.05; **P < 0.01; ***P < 0.001

To explore the mechanism of IL-27 in alveolar epithelial A549 cells, we first examined the effects of different concentrations of IL-27 (10 to 100 ng/ml) on the proliferation of A549 cells in an MTT assay. We found that the lowest effective concentration of IL-27 was 20 ng/ml, and 100 ng/ml was the best concentration (Fig. 1a). To ascertain the effects of various concentration of IL-27 on EMT-related changes in A549 cells, we examined the epithelial phenotype markers E-cadherin and the mesenchymal phenotypic marker of vimentin by western blot. IL-27 increased E-cadherin protein levels and decreased vimentin protein levels in a concentration-dependent manner (Fig. 1b). Using the same method, we ascertained that the lowest effective concentration of TGF-β1 was 40 ng/ml. And treatment with TGF-β1 at the concentration of 40 ng/ml for 48 h, there is almost one fold increase in the expression of E-cadherin and one fold decrease in vimentin protein levels. Thus, we used 100 ng/ml of IL-27 and 40 ng/ml of TGF-β1 for subsequent experiments.

### IL-27 inhibiting EMT in A549 cells

**Fig. 2:**
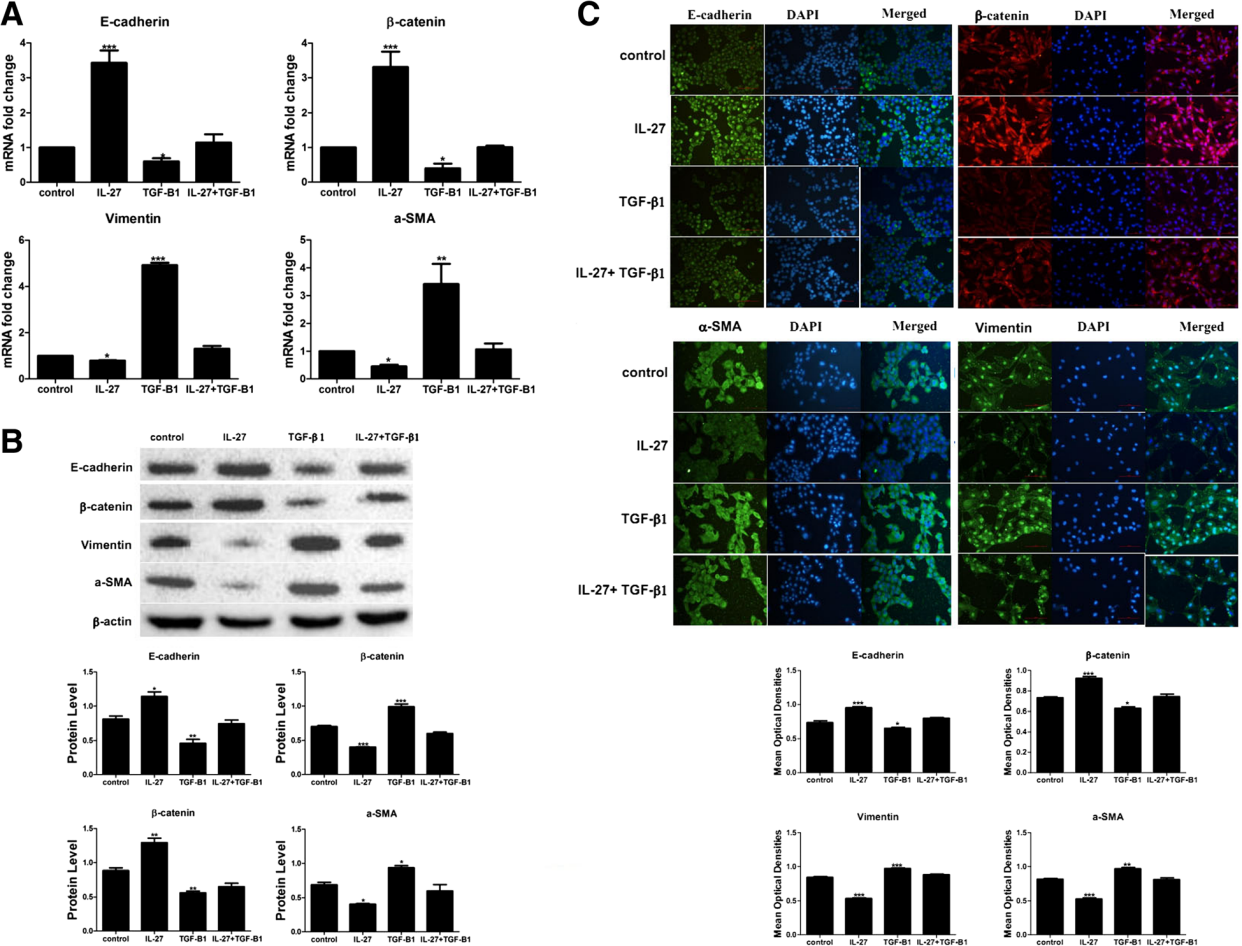
IL-27 attenuated TGF-β1-induced EMT in A549 cells. A549 cells were treated with 40 ng/ml of TGF-β1 or/and 100 ng/ml of IL-27 for 48 h. **a**, **b** The transcription levels of epithelial phenotypic markers E-cadherin and β-catenin and mesenchymal phenotypic markers vimentin and a-SMA were measured by real-time PCR and a western blot. **c** Fluorescence micrographs of E-cadherin, β-catenin, vimentin and α-SMA in AECs at 200X. Scale bars = 100 μm. Green or red indicates the protein of interest, and blue indicates the cell nucleus. Quantification of E-cadherin, β-catenin, vimentin and α-SMA in AECs was carried out using Image-Pro Plus 6.0 software. Mean optical densities were measured. All data are shown as the mean ± SD from at least three experiments. Statistical significance was assessed by one-way ANOVA and Tukey’s post hoc test. *P < 0.05; **P < 0.01; ***P < 0.001

To confirm the effect of IL-27 in alveolar epithelial cells, we first evaluated the transcription levels of E-cadherin, β-catenin, vimentin and a-SMA using real-time PCR. We found that IL-27 could enhance basal E-cadherin and β-catenin expression and decrease basal vimentin and a-SMA expression. TGF-β1 treatment of A549 cells for 48 h significantly reduced basal expression of E-cadherin and β-catenin and increased the basal expression of vimentin and a-SMA. E-cadherin and β-catenin levels in the presence of 40 ng/ml of TGF-β1 were increased by 100 ng/ml of IL-27, but vimentin and a-SMA levels were inhibited (Fig. 2a). To further examine the role of IL-27 in inhibiting EMT in alveolar epithelial cells, we performed western blots and immunofluorescence labelling to observe the protein expression level and obtained the same results as above (Fig. 2b, c). As the results shown, after exposure to IL-27 and TGF-β1 for 48 h, there is almost one fold increase in E-cadherin and β-catenin protein level and 0.5 fold decrease in vimentin and a-SMA expression compared to cells treated with TGF-β1 alone.

### IL-27 inhibits TGF-β1-induced EMT by inactivating the JAK/STAT signalling pathway

**Fig. 3:**
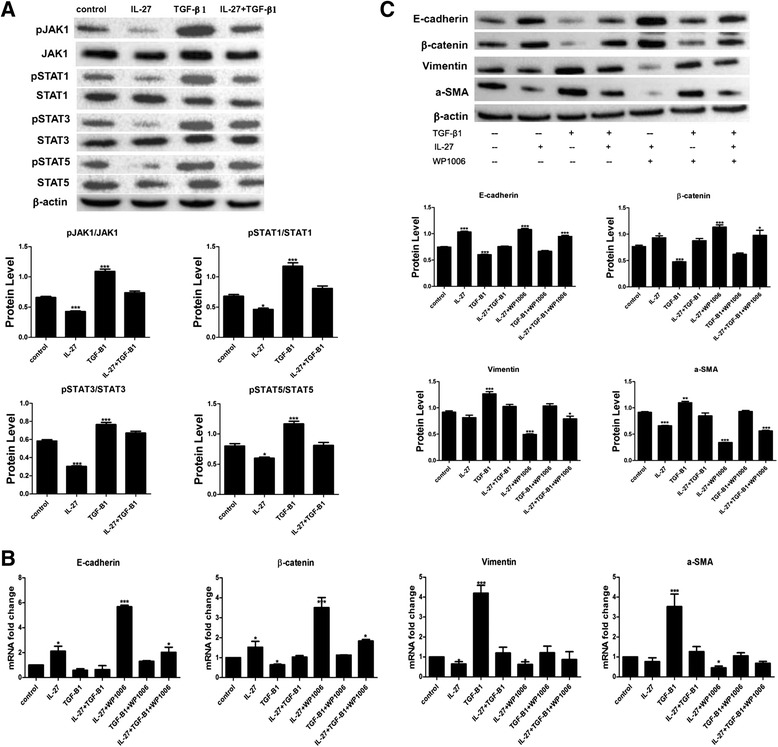
IL-27 inactivated the JAK/STAT signalling pathway in AEC. **a** JAK1, STAT1, STAT3 and STAT5 phosphorylated levels were measured by western blot after treatment with IL-27/and TGF-β1 for 1 h. **b**, **c** After exposure to a STAT3 specific inhibitor, E-cadherin, β-catenin, vimentin and α--SMA in AECs were measured by real-time PCR and western blot. All data are shown as the mean ± SD (*n* = 3). Statistical significance was assessed by one-way ANOVA and Tukey’s post hoc test. *P < 0.05; **P < 0.01; ***P < 0.001

To further explore the molecular mechanisms of IL-27 in EMT, we examined the expression of the JAK/STAT signalling pathway. After exposure to cytokines for 1 h, we found that TGF-β1-induced phosphorylation of JAK1, STAT1, STAT3 and STAT5 in AECs. When cells were treated concomitantly with IL-27 and TGF-β1, activation of JAK1, STAT1, STAT3 and STAT5 was partially inhibited, indicating that IL-27 modulates JAK1, STAT1, STAT3, and STAT5 phosphorylation (Fig. 3a). Furthermore, simultaneously treated AECs with the STAT3 specific inhibitor WP1006 could boost the inhibition effect of IL-27 on the mRNA and protein expression level of mesenchymal phenotypic markers vimentin and a-SMA and enhanced the expression of epithelial phenotypic markers E-cadherin and β-catenin compared with those treated with IL-27 alone (Fig. 3b, c). These above results indicated that the IL-27 inhibited EMT process partially through inactivating JAK/STAT signalling pathway.

### IL-27 affects TGF-β1-mediated phosphorylation of Smad1, Smad3 and Smad5

**Fig. 4:**
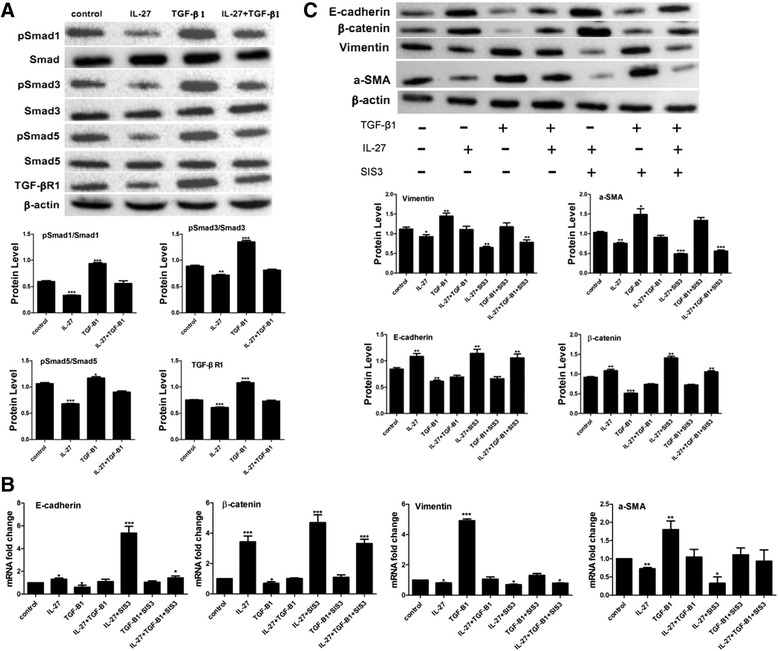
IL-27 affects TGF-β1-mediated EMT in AECs via the TGF-β1/Smad signalling pathway. **a** The protein levels of TGF-βR1, pSmad1, pSmad3, pSmad5 and their total protein were measured by western blot after treatment with IL-27/and TGF-β1 for 1 h. **b**, **c** A549 cells were treated with 40 ng/ml of TGF-β1 and 100 ng/ml of IL-27, a Smad3 specific inhibitor SIS3, or TGF-β1 and IL-27 and/or SIS3 for 48 h. Real-time PCR and western blotting was performed to analyse the expression of E-cadherin, β-catenin, vimentin and α-SMA. The results were quantified using Image J software. All data are shown as the mean ± SD (*n* = 3). Statistical significance was assessed by one-way ANOVA and Tukey’s post hoc test using GraphPad Prism Version 5.0a software. *P < 0.05; **P < 0.01; ***P < 0.001

TGF-β/Smad is a classical signalling pathway involved in TGF-β1-induced EMT. To determine whether IL-27 affects TGF-β1-induced EMT through the TGF-β/Smad signalling pathway, we investigated the expression of major proteins in the TGF-β/Smad signalling pathway after treatment with IL-27 and/ TGF-β for 1 h. The phosphorylation of Smad1, Smad3, Smad5 and the expression of TGF-βR1 were inhibited by IL-27 in the absence or presence of TGF-β1 (Fig. 4a). Moreover, we found that IL-27 could induce the expression of smad6 and smad7 and knocking down the expression of smad6 and smad7 could partially impair the function of IL-27 in reducing the phosphorylation of smad1/3/5(Additional file 1: Figure S1). Consistently, addition of the Smad3 specific inhibitor SIS3 weakened the effect of TGF-β1-induced EMT but strengthened the effect of IL-27 as indicated in the mRNA and protein expression level of mesenchymal phenotypic markers vimentin and a-SMA and epithelial phenotypic markers E-cadherin and β-catenin (Fig. 4b, c).

## Discussion

To further explore the mechanism of IL-27 in pulmonary fibrosis, we examined the role of IL-27 in EMT of A549 cells. We found that IL-27 could inhibit the EMT- related changes in A549 cells. Some studies have reported that TGF-β1 is a key regulator of EMT in pulmonary fibrosis; TGF-β1-induced EMT might be central to the process of collagen production and fibrosis. In fact, adding TGF-beta to epithelial cells in culture is a convenient way to induce EMT in various epithelial cells [11, 13–15]. Our results demonstrated that IL-27 co-cultured with TGF-β1 in AEC attenuated the EMT.

The regulation of TGF-β1-induced EMT is complex because TGF-β1 signalling occurs through different pathways, including Smads, mitogen-activated protein kinase (MAPK), and phosphatidylinositol 3-kinase (PI3K) pathways. Kolosova and colleagues demonstrated that both Smad2 and Smad3 were important for TGF-β1 function in cultured pulmonary epithelial cells [16]. Kasai proposed that the signalling pathway involved in alveolar EMT was likely to be a Smad2-dependent pathway. Our results also verified that TGF-β1 activated the phosphorylation of Smad1, Smad3 and Smad5, and IL-27 weakened the phosphorylated levels of these proteins in the process of inhibiting TGF-β1-induced EMT. Moreover, the Smad3 specific inhibitor SIS3 significantly strengthened the role of IL-27. These results suggest that IL-27 partially suppressed the TGF-β1-induced EMT in AEC through the TGF-β1/Smad signalling pathway. And inhibitory SMADs (SMAD6/7) as negative regulator of smad are involved in regulation of TGF-β pathway [17–19]. Our further results (Additional file 1: Figure S1) showed that IL-27 could induce the expression of smad6 and 7. And knocking down the expression of smad6 and smad7 by siRNA could partially impair the function of IL-27 in reducing the phosphorylation of smad1/3/5. These results suggest that IL-27 inhibiting the EMT partially through increase the function of inhibitory smads.

IL-27 is a member of the IL-12 family of cytokines and activates the JAK/STAT signal transduction pathway in a context-dependent manner [20]. In natives T cells, IL-27 induces T-Bet and IL-12Rbeta2 through Stat1 and Stat3 [21]. George proposed that IL-27 activates a STAT1-dominant pattern of signalling in human monocytes [22]. Kachroo demonstrated that IL-27 attenuated EMT and the production of pro-angiogenic factors in a STAT1-dominant pathway in human non-small cell lung cancer [23]. Yoshimoto proposed that IL-27 had an antiproliferative effect on melanomas through WSX-1/STAT1 signalling [24]. The aberrant activation of Stat occurs in many cancers [25]. Our results found that IL-27 could decrease the phosphorylation of STAT1, STAT3 and STAT5 during EMT. This is consistent with research done by Ko et al which previoulsy validated that activation of STAT3 could lead to decreased expression of E-cadherin [26]. Our results suggest that the JAK/STAT signalling pathway might be a key molecular mechanism of IL-27 activity in pulmonary fibrosis. Moreover, our results demonstrated that IL-27 attenuated the phosphorylated levels of JAK1, STAT1, STAT3 and STAT5 during EMT. However, we did not determine which STAT subtype is most predominant. And a further step needs to take to uncover why IL-27 could decrease the phosphorylation of STAT.

## Conclusion

In summary, the present study is the first report of the effects of IL-27 on EMT-related changes in A549 cells. Here, we show that IL-27 could inhibit TGF-β1-induced EMT and inactivate Smad and STAT signal transduction pathways in AECs. Our results increase the current understanding of IL-27 in EMT and identify new potential targets for therapeutic intervention of pulmonary fibrosis.

### Availability of data and materials

All the supporting data are included as additional files. Data are available in LabArchives, LLC (DOI: 10.6070/H45T3HHB).

## Additional file

Additional file 1: Figure S1. IL-27 affects TGF-β1-mediated EMT in AECs partially through inhibitory smads. A: The protein levels of smad6 and smad7, TGF-βR1, pSmad1, pSmad3, pSmad5 and their total protein were measured by western blot after treatment with IL-27/and TGF-β1 for 1 h. And statistically expression level of p-smad1, p-smad3 and p-smad5 was shown on the right, the relative expression level was compared with control and the change between TGF-β1 group and TGF-β1 + IL-27 group was also analysed. B: A549 cells were either left as control or transfected with siRNA specific to smad6 and smad7 for 6 h prior treated with 40 ng/ml of TGF-β1 and/or 100 ng/ml of IL-27. And the expression level of smad6 and smad7, TGF-βR1, pSmad1, pSmad3, pSmad5 and their total protein were measured by western blot in 4 different groups. And statistically expression level of p-smad1, p-smad3 and p-smad5 was shown on the right, the relative expression level was compared with control and the expression level between TGF-β1 + siRNA group and TGF-β1 + IL-27+ siRNA group was also analysed. All data are shown as the mean ± SD (n = 3). Statistical significance was assessed by one-way ANOVA and Tukey’s post hoc test using GraphPad Prism Version 5.0a software. *means compared with control group; # means TGF-β1 group compared with TGF-β1 + IL-27 group. **P* < 0.05; ***P* < 0.01; ****P* < 0.001; ##, *P* < 0.01; ###, *P* < 0.001, ns, no significant difference. (JPG 381 kb)

## Abbreviations

AECs: alveolar epithelial cells
EMT: epithelial–mesenchymal transition
IL-27: interleukin-27
JAK: Janus protein tyrosine kinase
MOD: mean optical density
QPCR: real-time quantitative PCR
STAT: signal transducer and activator of transcription
TGF-β1: transforming growth factor-β1
