# Supplementary figures and images for: IL-27 inhibits the TGF-β1-induced epithelial-mesenchymal transition in alveolar epithelial cells
Source: BMC Cell Biol. 2016 Mar 1;17:7. doi: 10.1186/s12860-016-0084-x (PMC4774182; doi:10.1186/s12860-016-0084-x)

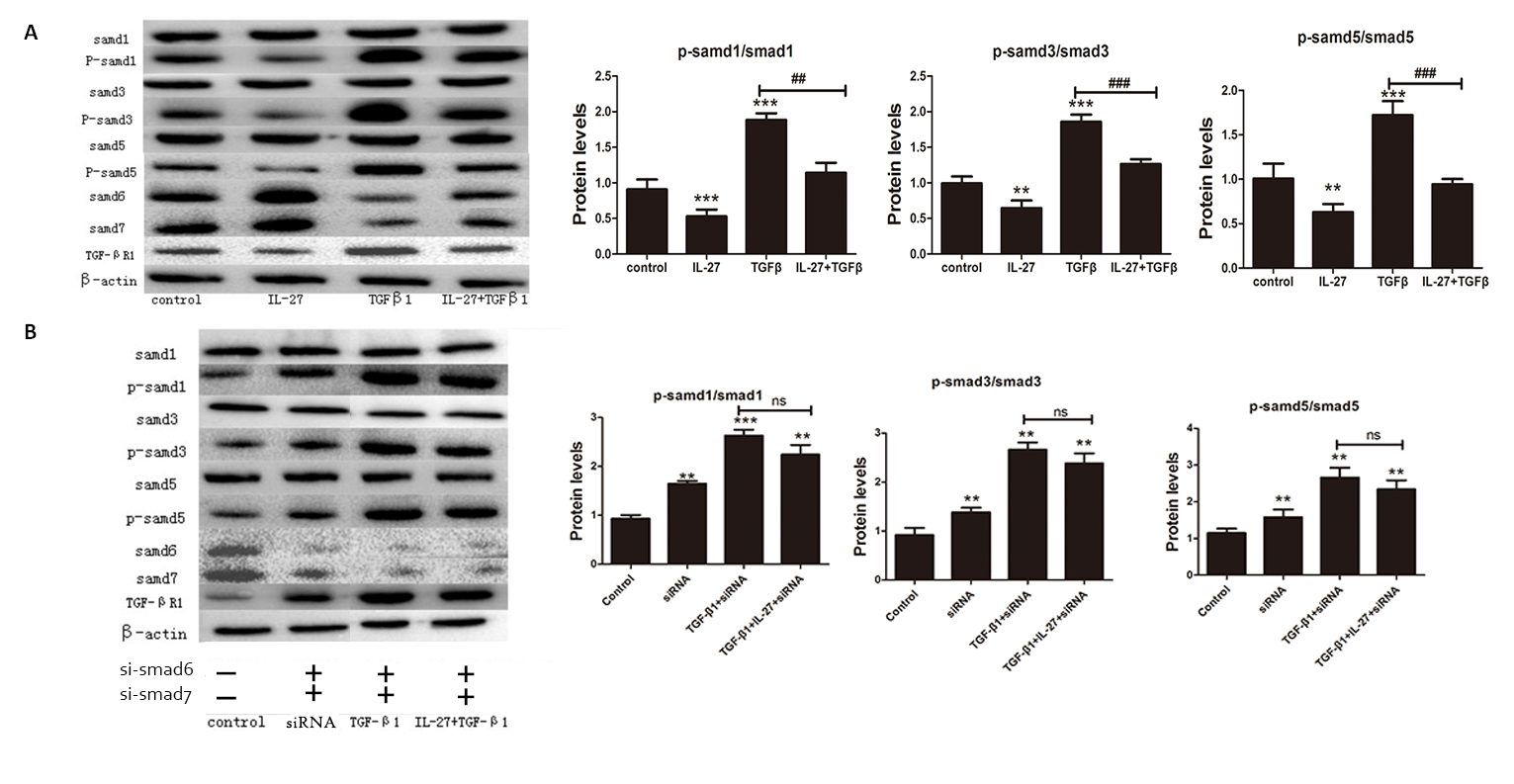

Supplement: Additional file 1: Figure S1. — IL-27 affects TGF-β1-mediated EMT in AECs partially through inhibitory smads. A: The protein levels of smad6 and smad7, TGF-βR1, pSmad1, pSmad3, pSmad5 and their total protein were measured by western blot after treatment with IL-27/and TGF-β1 for 1 h. And statistically expression level of p-smad1, p-smad3 and p-smad5 was shown on the right, the relative expression level was compared with control and the change between TGF-β1 group and TGF-β1 + IL-27 group was also analysed. B: A549 cells were either left as control or transfected with siRNA specific to smad6 and smad7 for 6 h prior treated with 40 ng/ml of TGF-β1 and/or 100 ng/ml of IL-27. And the expression level of smad6 and smad7, TGF-βR1, pSmad1, pSmad3, pSmad5 and their total protein were measured by western blot in 4 different groups. And statistically expression level of p-smad1, p-smad3 and p-smad5 was shown on the right, the relative expression level was compared with control and the expression level between TGF-β1 + siRNA group and TGF-β1 + IL-27+ siRNA group was also analysed. All data are shown as the mean ± SD (n = 3). Statistical significance was assessed by one-way ANOVA and Tukey’s post hoc test using GraphPad Prism Version 5.0a software. *means compared with control group; # means TGF-β1 group compared with TGF-β1 + IL-27 group. *P < 0.05; **P < 0.01; ***P < 0.001; ##, P < 0.01; ###, P < 0.001, ns, no significant difference. (JPG 381 kb) [file 12860_2016_84_MOESM1_ESM.jpg]
